# Supplementary material for: Identification of photoperiod-induced specific miRNAs in the adrenal glands of Sunite sheep (Ovis aries)
Source: Front Vet Sci. 2022 Jul 22;9:888207. doi: 10.3389/fvets.2022.888207 (PMC9354845; doi:10.3389/fvets.2022.888207)
Supplement: Supplementary file 1 [file Data_Sheet_1.ZIP › Supplementary file/miRDeep2 file.zip!/miRDeep2 file/Know_Structure/SPLP42A1/oar-mir-107.pdf]

miRBase precursor	: oar-mir-107
Total read count	: 5752
oar-miR-107 read count	: 5748
remaining reads	: 4

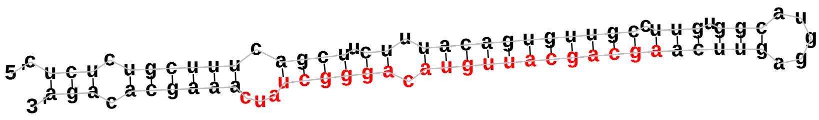

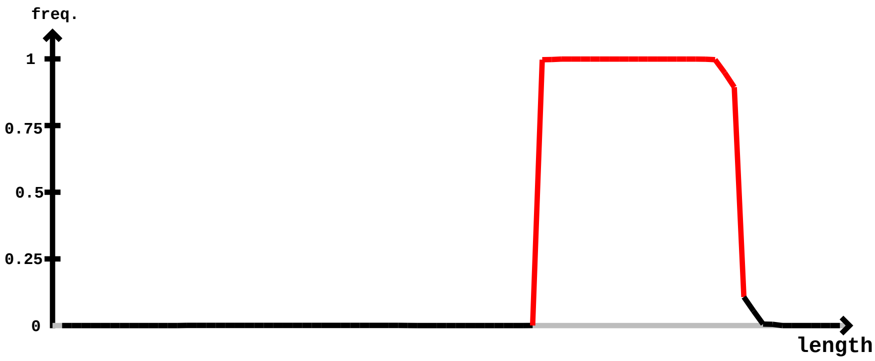

oar-miR-107

```
5'- cucucugcuuucagcuucuuuacaguguugccuuguggcauggaguucaagcagcauuguacagggcuaucaaagcacaga   -3'    exp
    .(((.((((((.((((.((.(((((((((((.(((.(((.....))))))))))))))))).))))))...)))))).)))    reads      mm         sample
    ............agcuucuuuacaguguugccuug..............................................    1          0          seq
    ............agcuucuuuacagugCugccuug..............................................    1          1          seq
    ............agcuucuuuacagugCugccuugu.............................................    2          1          seq
    ................................................aagcagcauuguacagggcuau...........    1          0          seq
    ................................................aagcagcauuguacagggcuauc..........    1          0          seq
    .................................................agcagcauuguacaggg...............    3          0          seq
    .................................................agcagcauuguacagggc..............    10         0          seq
    .................................................agcagcauuguacagggcu.............    280        0          seq
    .................................................agcagcauuguacagggcua............    312        0          seq
    .................................................agcagcauuguacagggcuau...........    4516       0          seq
    .................................................agcagcauuguacagggcuauc..........    298        0          seq
    .................................................agcagcauuguacagggcuauca.........    284        0          seq
    .................................................agcagcauuguacagggcuaucaa........    5          0          seq
    .................................................agcagcauuguacagggcuauGaaa.......    24         1          seq
    ..................................................gcagcauuguacagggcu.............    1          0          seq
    ..................................................gcagcauuguacagggcuau...........    3          0          seq
    ...................................................cagcauuguacagggcuau...........    6          0          seq
    ...................................................cagcauuguacagggcuauca.........    1          0          seq
    ...................................................cagcauuguacagggcuauGaaa.......    3          1          seq
```
